# Slipping mechanics during walking along curved paths depend on the biomechanical context at slip onset

**DOI:** 10.1038/s41598-022-21701-7

**Published:** 2022-10-23

**Authors:** Corbin M. Rasmussen, Carolin Curtze, Mukul Mukherjee, Nathaniel H. Hunt

**Affiliations:** Department of Biomechanics, University of Nebraska at Omaha, Omaha, NE 68182 USA

**Keywords:** Biomedical engineering, Rehabilitation, Motor control

## Abstract

Curvilinear walking is common, causing limb- and radius-dependent asymmetries that distinguish it from straight walking and elevated friction demands that increase slip-and-fall risk. However, it is unclear how aspects of curvilinear walking influence the slip perturbations experienced. We cross-sectionally examined how three biomechanical slip contexts (slip onset phase, slipped foot relative to the path, path radius) influence slip direction, distance, and peak velocity. Eighteen young adults experienced unconstrained inside or outside foot slips during early, mid-, or late stance while following 1.0- or 2.0-m radius semicircular paths. We derived slip mechanics from motion-capture data and assessed their dependence on slip context using mixed-effects models. As slip onset phase progressed, slip directions exhibited an anterior-to-posterior transition, shortened mediolaterally, and accelerated anteroposteriorly. The slipped foot modified the direction transition, with inside and outside foot slips moving contralaterally and ipsilaterally, respectively. Inside foot slips were shorter and slower mediolaterally and longer anteroposteriorly than outside foot slips. Increasing path radius caused slips with greater mediolateral direction components. We show a range of context-dependent slips are possible, likely due to instantaneous magnitudes and orientations of shear ground reaction forces. Our results contribute to a comprehensive understanding of walking slips, which fall prevention methods can leverage.

## Introduction

Falls are a significant public health problem, ranking among the leading causes of injury^1^ and death^2^ in all adult age groups. Extrinsic events such as slips while walking contribute a majority of reported falls in the community and can happen under a broad range of contexts and conditions^3,4^. One particular form of gait that may prove essential to a comprehensive understanding of slip-related falls is curvilinear walking, which constitutes up to 45% of our daily steps^5^. A number of biomechanical aspects distinguish curvilinear from straight walking. Curvilinear walking is characterized by large shear ground reaction forces (GRFs) and impulses directed outward from the center of the turn, regardless of which foot is in stance (i.e. contralaterally/ipsilaterally directed shear GRFs under the inside/outside foot relative to the turn, respectively)^6,7^. As a result, curvilinear walking requires an elevated coefficient of friction compared to straight, level walking in younger adults^8^ but not necessarily in healthy older adults^9,10^ due to a reduced lateral lean angle^10^. Muscle activations are generally delayed in the inside leg and advanced in the outside leg relative to the path, with activation magnitudes correlating with the path radius^11,12^. Based on musculoskeletal simulation, the inside leg undergoes more drastic muscle coordination adjustments to change heading direction than the outside leg^13^. Finally, spatiotemporal attributes are altered during curvilinear walking, with the inside leg taking shorter, narrower, longer lasting steps^12,14^. Altogether, asymmetry between lower limbs is a fundamental property of curvilinear walking, determined by the direction and rate at which one is altering their heading.

Despite the fact that curvilinear walking is biomechanically distinct from straight walking, few published studies have examined slips during this form of gait. Those that have reported increasing slip severity and fall risk with tighter turn angles^15^, that the outcome of a slip (i.e. fall vs. recovery) can be predicted by the distance separating the center of pressure and a zero moment point^16^, and that turning slips were primarily directed forward at a faster velocity than those during straight walking, gait initiation, and termination^17^. While these findings justify further study of slips during curvilinear walking given the heightened slip severity and fall risk^15,17,18^, it is still unclear how these balance perturbations are influenced by the unique contexts present during the task, such as the path radius and the foot relative to the path that loses traction.

A primary reason for the lack of slip research on curvilinear paths may be the lack of viable, laboratory-based perturbation methods that are spatially unconstrained, allowing realistic slips to be induced while one changes their heading direction^19,20^. All three studies cited previously used lubricated floors to elicit slips^15–17^, however the predictability of this technique introduces anticipatory adjustments that may influence the results^20^. Treadmills and sliding platforms, while invaluable tools for emulating slips during straight walking, cannot mimic non-straight gait. In order to properly study curvilinear walking slips, a method that minimizes predictability while delivering mechanically unrestricted perturbations during over-ground walking would be ideal. Recently, we developed a wearable slipping device that meets these criteria^21^. This device can be activated to suddenly reduce the available friction underfoot at any point during stance, allowing for the effect of slip onset timing during stance phase to be examined. In a previous study of slips during straight walking, slip mechanics and compensatory stepping behaviors were greatly influenced by slip onset timing within stance^22^. However, because it is biomechanically distinct from straight walking, these results cannot be generalized to slips occurring on curvilinear paths.

Using our slipping device, the purpose of this study was to determine how various biomechanical slip contexts shape slips experienced on semicircular paths used to elicit a constant, curvilinear gait. Based on the literature and the capabilities of our device, the specific contexts we chose to manipulate were path radius, slipped foot relative to the path, and slip onset phase within stance, while the slip mechanics we chose to examine were direction, distance, and peak velocity. We hypothesized that slip directions relative to heading would grow (in terms of the angle with heading) with later slip onset phases, shrink with increasing path radius, and interact with the slipped foot relative to the path. Second, we hypothesized that slip distances in both the anteroposterior (AP) and mediolateral (ML) planes would decrease with later slip onset phase and larger path radius as well as be shorter after inside foot slips compared to outside foot slips. Finally, we hypothesized that peak slip velocity in both AP and ML planes would slow with later slip onset phase, larger path radius, and inside foot slips relative to outside foot slips.

## Methods

### Participant recruitment

Eighteen healthy young adults (age: 22.72 ± 2.89 years, height: 1.73 ± 0.09 m, mass: 72.25 ± 12.35 kg, 9 females) provided written informed consent and were screened via questionnaire for participation in the present study. Exclusion criteria included any musculoskeletal, neurological, and cardiopulmonary conditions that may influence their otherwise natural gait patterns, the use of an assistive device such as a cane, walker, prosthetic, or orthotic; uncontrolled hypertension, pregnancy, joint or muscle pain, and a history of injurious falls beyond contusions, lacerations, and abrasions. After obtaining their written consent, each participant’s height and mass were measured and recorded, and limb dominance was self-reported through the screening questionnaire. All study procedures were approved by the Institutional Review Board of the University of Nebraska Medical Center (IRB#: 227-18-FB), performed in accordance with the Declaration of Helsinki, and completed in a single laboratory visit.

### Device design

**Figure 1:**
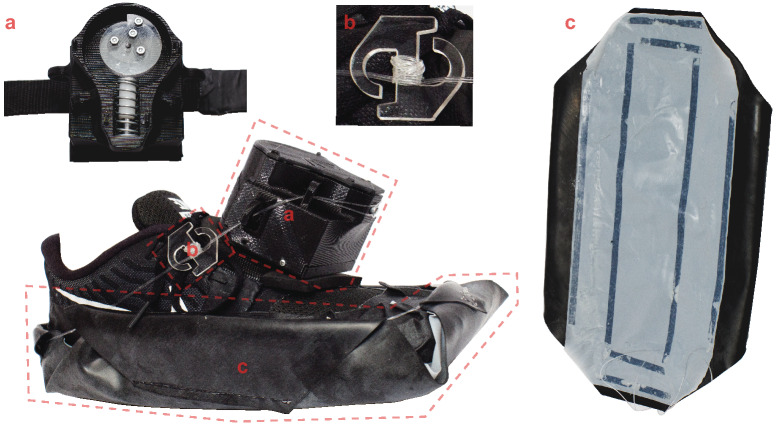
The Wearable Apparatus for Slip Perturbations (WASP) used in this study. This device consists of three components: (**a**) a wireless triggering mechanism consisting of a motorized cam-and-follower, (**b**) a tensioner that allows the device to fit securely to a range of shoe sizes, and (**c**) a flexible rubber outsole laminated with a polytetrafluoroethylene sheet that supplies both the friction with the ground to walk naturally in its attached state and the low-friction surface that participants are exposed to upon triggering. Lubricant was applied between the shoe and the outsole between each trial to further reduce friction upon outsole release.

We recently developed a new slip perturbation device called the Wearable Apparatus for Slip Perturbations (WASP, Fig. 1), the function of which has been described in detail elsewhere^21^. Briefly, the rubber outsole component of the device supplies adequate friction with the ground until triggered by the researcher via wireless connection, which suddenly exposes the wearer to a very low friction surface created by lubricated layers of polytetrafluoroethylene (i.e. Teflon™) between the outsole and wearer’s shoe. The version of WASP used in the present study is refined from that reported by Rasmussen and Hunt in 2019^21^. First, we used an alternative cam-and-follower release mechanism, which was also described in the discussion section of that paper. This mechanism reduces the mechanical delay between activation by the investigator and WASP outsole release, allowing more accurately timed slips to be delivered. Second, a thin rubber sheet was used for the outsole component, which decreases the device’s overall weight and provides a more secure fit to the wearer’s shod foot due to its pliability. While certain design aspects of the device used here are different from what was previously published, the overall function is the same.

### Experimental design

**Figure 2:**
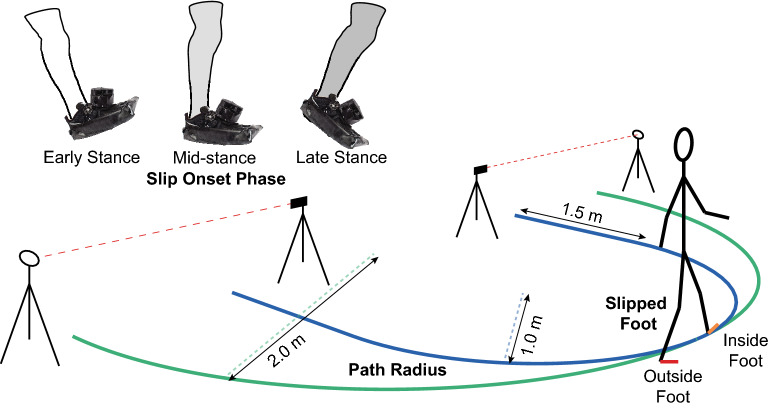
An illustration of the experimental set-up used in this study. 1.0 m and 2.0 m radius paths (blue and green paths, respectively) were marked on the floor with different colors of paper tape for easy identification, and the paths were tangent at their apexes. Straight sections of 1.5 m were added to both ends of the 1.0 m radius path to approximately equalize the lengths of the two paths and allow participants to accelerate to their self-selected walking speed before entering the curve. Participants walked back-and-forth along one path in each trial, following the curve with their inside foot relative to the path to ensure a natural step width and to place the CoM near the intended radius. A slip was delivered during each trial via WASP to either the inside or outside foot relative to the path (orange and red feet, respectively) at early, mid-, or late stance phases (white, light gray, and dark gray, respectively). Electronic timing gates spanned both ends of the paths as a method of measuring gait speed in real time for feedback purposes. Color coding of the three slip contexts in the results are consistent with this figure.

All participants were outfitted with a form-fitting compression suit, fall-arresting safety harness, lab-provided athletic shoes in their size, plantar pressure insoles (Pedar-X, Novel GmbH; Munich, Germany) matched to their shoe size, and a full-body, 79-marker retroreflective marker set prior to beginning the study. Marker trajectories were collected at 200 Hz using a 17-camera motion capture system (Motion Analysis Corp.; Santa Rosa, CA), and plantar pressure data were sampled at 100 Hz. A second wireless receiver was also connected to the motion capture system in order to record WASP trigger times in synchrony with the kinematic data. Two semicircular paths of different radii (1.0 m and 2.0 m, Fig. 2) were marked on the floor of the lab in different colors of paper tape for easy identification. Each path encompassed 180°, with the 1.0-m path internally tangent to the 2.0-m path at their apexes. At both ends of the 1.0-m path, a straight segment was added to allow participants to accelerate before entering the curve as well as to equalize the lengths of the two paths. Participants were instructed to follow the marked paths with their inside foot relative to the path. The reason for this was twofold: (1) to prevent the adoption of an unnaturally wide gait or a zero-width gait as a result of participants straddling the line or stepping on the line with every step, respectively, and (2) at a comfortable, self-selected speed, the center of mass (CoM) tracks directly above the inside foot, placing it very near the intended radius of the path^6^. Electronic timing gates (Dashr, LLC; Lincoln, NE) were placed at both ends of the paths.

### Experimental protocol

At any time when the participants were not seated, their worn safety harness was connected to a ceiling-mounted trolley system and adjusted to prevent contact with the ground in the event of a fall. Each participant was allowed a familiarization period to the semicircular paths consisting of six passes on each at the participant’s self-selected comfortable speed. The speed of each pass on both paths was measured in real time using the timing gates, then averaged together to obtain a mean comfortable gait speed unique to each path. Once this familiarization period was completed on both paths, a 3-min seated rest period was given to each participant while the attending investigators attached a WASP device (Fig. 1) to both of their shod feet.

All study participants performed 12 unique slipping trials, the order and duration of which was randomized prior to each session. Each trial exposed the participant to a different combination of the three slip contexts, which were the timing of the slip during stance phase of the slipped foot (early, mid-, or late stance; Fig. 2), the slipped foot relative to the path (inside or outside; Fig. 2), and the path radius (1.0 m or 2.0 m; Fig. 2). Early stance was defined as 0–33.3%, mid-stance as 33.4–66.7%, and late stance as 66.8–100% of stance phase. Participants always started at the same end of the prescribed path, followed it to the other end, then turned completely around and followed it back to their starting point. This back-and-forth routine continued in every trial until the individual was instructed to stop. Participants walked at the mean comfortable speed for each path that was observed during the familiarization period, and verbal feedback from an investigator was given to the participant when necessary to maintain that speed. Each trial lasted between 30 s and 3 min. After the prescribed time for each trial elapsed, an attending researcher remotely triggered one WASP device on the appropriate foot targeting the intended phase of stance. A 3-min seated rest was allowed between each repetition to allow for reattachment of the triggered WASP device.

### Data analysis

Marker trajectories were filtered using a 4th order, zero-lag Butterworth filter with a cutoff frequency of 6 Hz. Because our participants did not walk along a principal axis of the lab, movements in the AP and ML planes were not easily derived using the lab reference frame. To calculate our slip mechanics of interest in an intuitive, egocentric reference frame, a virtual lab segment was created in Visual3D (C-Motion, Inc.; Germantown, MD). The origin of this virtual lab segment was the same as the global origin, but referenced the orientation of the pelvis segment to define the X-axis (i.e. the pelvis X-axis and virtual lab X-axis were parallel). This caused the virtual lab to rotate along with the participant as they changed their heading, meaning that the virtual lab Y-axis was always aligned with the participant’s heading and the X-axis orthogonal to that and the vertical.

A coordinate-based method was employed to determine heel strike and toe-off events from kinematic data^23^. Using these gait events as a reference, the actual slip onset time in each trial was calculated from the velocity of the slipped foot segment’s CoM. The time of slip onset was defined as either the instant that the slipped foot’s CoM velocity exceeded zero following heel strike or, in the case that the velocity never reached zero after heel strike, at heel strike. The time between heel strike and slip onset was then calculated and divided by the average stance duration of that condition set to obtain the slip onset phase. Onset phases were then binned into the three phases of interest, and the result of this calculation and binning is presented in the “Slip Occurred” column of Table 1. The instant of slip cessation was also determined from the slipped foot’s CoM velocity, specifically when the velocity returned to zero after slip onset or, if this never happened before toe-off of that foot, at the instant of toe-off. WASP trigger phases were derived and binned in a similar way as slip onset phases: the time between WASP triggering as measured by the receiver connected to the motion capture system and heel strike of the targeted stance phase was calculated and divided by the average stance duration within that same condition set, then sorted into the three phases of interest. In cases where WASP was triggered prior to heel strike of the targeted stance phase, the trial was classified as early stance. The result of WASP trigger phase calculation and binning is presented in the “WASP Triggered” column of Table 1. Many trials consisted of WASP triggering during an earlier stance phase than that at which slip onset actually occurred, evidenced by the discrepancy between “WASP Triggered” and “Slip Occurred” trial counts in Table 1. To convey this, the trials within each phase where friction was reduced by WASP prior to a slip occurring were counted and reported in the “Friction Reduced” column of Table 1. For example, a trial where WASP was triggered during early stance but did not cause a slip until late stance was counted in all three stance phase bins under “Friction Reduced”. Finally, the probability that a trial ended with a slip when friction was reduced was calculated by dividing the total number of slips within each condition set (Slip Occurred) by the number of times friction was reduced within that same condition set (Friction Reduced) and reported in the “Slip Probability” column of Table 1. It is important to note that these probabilities do not sum to 100% because they represent the observed likelihood of a slip *within* each phase if friction underfoot was reduced, not the observed likelihood across all phases within a given slipped foot/path radius condition set.

**Table 1:** Descriptive details about the slip perturbations delivered in this study.

Path radius	Slipped foot	Slip onset time	WASP triggered (n)	Friction reduced (n)	Slip occurred (n)	Slip probability (%)
1.0 m (n = 108)	Inside (n = 53)	Early stance	46	46	12	26.1
		Mid-stance	6	40	2	5.0
		Late stance	1	39	11	28.2
	Outside (n = 55)	Early stance	48	48	36	75.0
		Mid-stance	6	18	9	50.0
		Late stance	1	10	6	60.0
2.0 m (n = 108)	Inside (n = 54)	Early stance	48	48	18	37.5
		Mid-stance	6	36	0	0.0
		Late stance	0	36	18	50.0
	Outside (n = 54)	Early stance	47	47	26	55.3
		Mid-stance	7	28	8	28.6
		Late stance	0	20	13	65.0

WASP trigger phases were calculated as the time between WASP triggering and heel strike of the targeted stance phase divided by the mean stance duration within a given condition set, then binned into early, mid-, and late stance phases (WASP Triggered). “WASP Triggered” values here are trial counts within each phase bin. The number of trials within each condition set where friction was reduced prior to a slip occurring is given in the “Friction Reduced” column. Note that these are cumulative counts including trials where friction was reduced by WASP triggering at an earlier stance phase than when a slip actually occurred. For example, a trial where WASP was triggered during early stance but the resulting slip occurred during late stance would be counted in early, mid-, and late stance bins under “Friction Reduced”. Slip onset phases were calculated as the time between slip onset and heel strike of the targeted stance phase divided by the mean stance duration of that condition set and binned into the three stance phases, presented here as trial counts within each bin (Slip Occurred). These three columns are ordered temporally: WASP triggering occurs first, causing friction reduction underfoot that potentially, whether within the same or later phase of stance, results in a slip. Finally, “Slip Probability” was calculated by dividing the “Slip Occurred” value by the “Friction Reduced” value for a given condition set, returning the likelihood that a slip would occur if friction was reduced under those conditions.

Slip mechanics were also derived from kinematic data. Slip directions were taken as the angle projected on the horizontal plane between the whole-body CoM heading and the slipped foot’s heading between slip onset and cessation. These directions were calculated in a range of 0° to 360°, with 0°/360° representing anteriorly-directed slips, 90° representing ipsilaterally-directed slips, 180° representing posteriorly-directed slips, and 270° representing contralaterally-directed slips. Slip distances were calculated as the displacement of the slipped foot’s CoM between slip onset and cessation, and peak slip velocities were taken from the same time period. Slip distances and peak velocities were subsequently reduced to their AP and ML components before analysis.

### Statistical analysis

Despite an equal number of *attempted* slips under every combination of contexts, we expected unequal numbers of *successfully delivered* slips across these conditions due either to some trials ending without a loss of traction or to manual WASP activation at the incorrect onset phase. To account for an unbalanced dataset across conditions, mixed-effects model analyses were used to examine the influence of slip context (i.e. onset phase, slipped foot, and path radius) on the resulting slip mechanics (i.e. direction, distance, and peak velocity). Mixed-effects models are robust to data that are “missing at random” as in this study, where the fact that a data point is missing is related to observed data (i.e. slip context) but not unobserved data (i.e. slip mechanics outcome measures). In all models, slip onset phase (3 levels; early stance was the default level for this predictor), slipped foot (2 levels; outside foot was the default level for this predictor), and path radius (2 levels; 1.0-m radius was the default level for this predictor) were entered as fixed effects. Participant was entered as a random effect to resolve dependencies within the fixed effects due to the repeated measures study design (Eq. 1).

1 $$\text{Slip Outcome} \sim \text{Phase + Foot + Radius + Phase*Foot + }\left(\frac{1}{{\text{Participant}}}\right)$$

Because the slip direction data were periodic in nature, directional statistics were used to examine how slip contexts influence slip direction. A Bayesian circular mixed-effects model (Eq. 1) was built in RStudio (RStudio, PBC; Boston, MA) based on the work of Cremers and colleagues^24,25^ using the R package “bpnreg”^26,27^. Input parameters (i.e. number of iterations, burn-in period, and lag) were tuned iteratively to ensure the Markov Chain Monte Carlo sampler converged on reliable coefficient estimates^28,29^. The resulting bivariate estimates of the tuned model were then transformed to univariate coefficient estimates interpretable on the circle^25^. Additional details on the design, tuning, and treatment of the circular mixed effects model and its results are provided in Supplementary Methods and Supplementary Figs. S1–S4 online. Significant effects of slip context were determined from the 95% highest posterior density (HPD) intervals for each context’s coefficient estimate: if a 95% HPD interval for a given coefficient estimate did not contain 0, then we concluded that the context in question had a significant effect on slip direction^24^. Note that the coefficient estimates returned by the circular model are relative values to the default set of independent variable levels (i.e. early stance, outside foot, 1.0-m radius path), thereby representing the change in slip direction from the default condition set exerted by each non-default independent variable level. Because slip onset phase had more than two levels, the 95% HPD intervals between mid-stance and late stance slip onset phases were also compared.

Linear mixed-effects models were applied in MATLAB (The MathWorks, Inc.; Natick, MA) to assess how slip contexts influence slip distance and peak velocity (Eq. 1). Separate models were built for the AP and ML components of both slip distance and peak velocity. As in the circular model described above, the coefficient estimates from these linear models are relative values to the default condition set. The explained variance of each linear model was assessed using the adjusted coefficient of determination (R^2^), and the critical alpha for all models and tests was set to α = 0.05.

## Results

Our study participants self-selected walking speeds of (mean ± SD) 1.00 ± 0.14 m/s on the 1.0-m radius path and 1.17 ± 0.15 m/s on the 2.0-m radius path during the familiarization period. As mentioned in the “Experimental protocol” section of the Methods section, participants were held to their personal average self-selected walking speed on a given path radius during all ensuing slip trials on that path radius.

### Slip distribution across contexts

**Figure 3:**
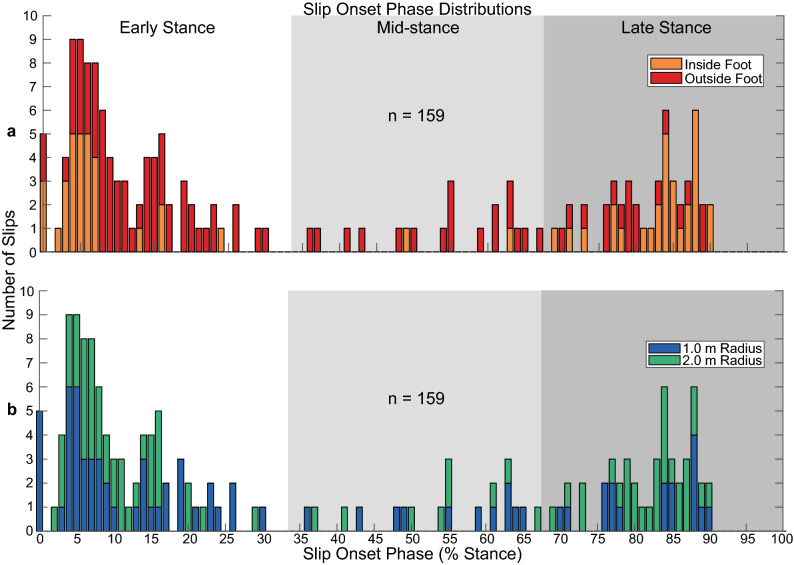
The distribution of successfully delivered slips across stance phase, color-coded by (**a**) slipped foot relative to the path and (**b**) path radius. Bars at 0% stance phase represent trials where the slipped foot’s CoM velocity never reached zero after heel strike and therefore slip onset occurred simultaneously with heel strike. Successfully delivered slips were concentrated in early and late stance, and those that did occur during mid-stance were almost exclusive to the outside foot.

A total of 216 slip perturbations were attempted over the course of this study (Table 1). Of these, 57 trials (26.4% of *total attempted* trials) did not result in an actual slip and were excluded from further analysis. Of those 57 unsuccessful trials, 46 (80.7% of *unsuccessful* trials) were intended to perturb the inside foot relative to the path. This exclusion left 159 trials (73.6% of *total attempted* trials) with successfully administered perturbations. A broad, bimodal distribution of slip onset phases was obtained (Fig. 3). Far fewer mid-stance slips occurred than both early and late stance slips (Fig. 3), and those that did were almost exclusively delivered to the outside foot relative to the path (17 outside foot vs. 2 inside foot; Fig. 3a). This non-uniform slip distribution across stance was not simply the result of a non-uniform distribution of WASP trigger phases, despite a disproportionate number of trials *triggered* before or during early stance. WASP trigger phases often did not correspond to slips occurring within the same phase, leading to a more uniform distribution of friction reduction across the three phases of interest. Despite this fact, slips were generally less likely to occur during mid-stance than during early or late stance in normalized terms as well (Table 1). Of the successfully delivered slips, 20 trials (9.3% of *total attempted* trials) were missing marker trajectories at either the foot or pelvis that prevented the calculation of our outcome measures. These trials were also excluded from further analysis. As a result, 139 successful, complete slip trials (64.4% of *total attempted* trials) were fully analyzed and included in the results that follow.

### Slip direction

**Figure 4:**
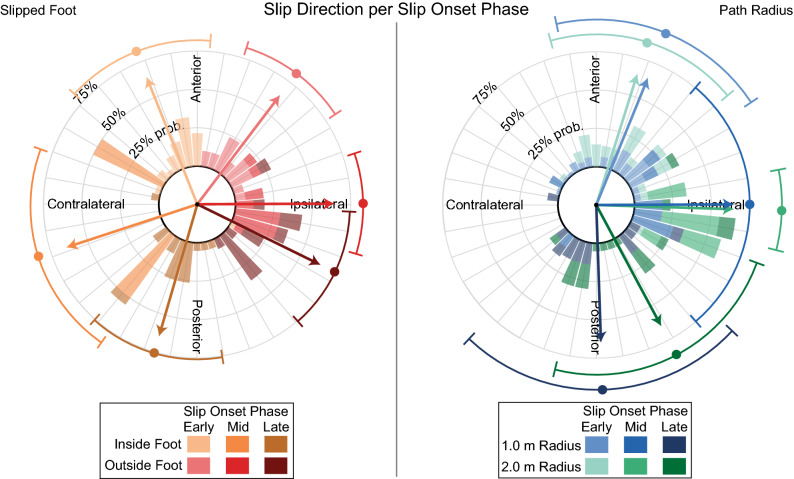
Slip direction relative to the heading of the CoM, grouped by onset phase-slipped foot and onset phase-path radius. Each bin is normalized to the total number of successfully delivered slips within the corresponding onset phase-slipped foot or onset phase-path radius combination, representing the probability that a slipped foot under each combination traveled in that direction. Each bar and angular grid line represents a 10° bin. Contralateral and ipsilateral directions are defined relative to the slipped foot. While these directions are presented in an egocentric reference frame, it is important to note that contralateral inside foot and ipsilateral outside foot slips are both directed outward from the semicircular path in an allocentric reference frame. Arrows within and points outside of the plots represent the mean direction within each combination of conditions, while error bars outside of the plots depict the corresponding circular standard deviations.

Slip direction was significantly predicted by the slipped foot (95% HPD: 323.4°–341.6°; Table 2) and slip onset phase (mid-stance 95% HPD: 78.9°–101.2°; late stance 95% HPD: 106.6°–138.0°; Table 2). A significant interaction was found between late stance slip onset and slipped foot (95% HPD: 17.8°–123.7°; Table 2), but not between mid-stance onset and slipped foot (95% HPD: 249.9°–103.0°, this interval contains a direction of 0°; Table 2). As slip onset progressed through stance phase, slip directions transitioned from predominantly anterior to predominantly posterior (circular median early stance: 23.8°, mid-stance: 91.3°, late stance: 173.7°; Fig. 4). This transition moved ipsilaterally (i.e. away from the body) in the case of outside foot slips during late stance, but contralaterally (i.e. across the body) for inside foot slips during late stance (circular median: 125.2° vs. 192.4°, respectively; Fig. 4). A similar foot-dependent transition is visible in the limited data for mid-stance slips (circular median: 91.3° vs. 252.4°, respectively; Fig. 4), however the interaction was not significant. The radius of the path also significantly influenced slip direction (95% HPD: 33.6°–46.8°; Table 2), indicating that slips occurring on the 2.0-m radius path tended to travel at a larger angle with the heading direction than those on the 1.0-m radius path (circular median: 56.2° vs. 44.5°, respectively; Fig. 4).

**Table 2:** Results from the circular and linear mixed-effects models generated for each slip outcome variable.

Coefficients	Linear coefficients (bivariate cartesian coord.)						Circular coefficients (deg.)				
	Mean I	95% HPD		Mean II	95% HPD		Mean	Mode	SD	95% HPD	
		Lower	Upper		Lower	Upper				Lower	Upper
**Slip direction**											
**Intercept**	**2.41**	**1.88**	**2.94**	**1.66**	**1.23**	**2.11**	**34.57**	**34.28**	**3.30**	**28.13**	**41.01**
**Path radius**	0.14	− 0.34	0.63	**0.47**	**0.02**	**0.93**	**39.96**	**39.97**	**3.37**	**33.62**	**46.77**
**Slipped foot**	0.36	− 0.60	1.23	**− 3.10**	**− 3.72**	**− 2.45**	**332.51**	**333.41**	**4.65**	**323.39**	**341.58**
**Mid-stance onset**	**− 2.43**	**− 3.14**	**− 1.73**	**1.66**	**0.45**	**2.95**	**90.37**	**90.94**	**5.63**	**78.87**	**101.20**
**Late stance onset**	**− 3.71**	**− 4.59**	**− 2.85**	0.46	− 0.53	1.49	**121.72**	**121.32**	**7.95**	**106.57**	**137.96**
Foot-mid interaction	− 0.86	− 2.85	1.17	− 1.72	− 4.07	0.40	357.49	356.44	48.95	249.93	103.02
**Foot-late interaction**	**− 1.69**	**− 3.10**	**− 0.24**	0.06	− 1.15	1.3	**68.34**	**63.07**	**27.07**	**17.83**	**123.66**

Coefficients	Estimate	SE	t(132)	p-value	95% C.I		Adj. R^2^	F(6,132)	p-value		
					Lower	Upper					
**AP slip distance (m)**											
**Intercept**	**0.074**	**0.002**	**4.72**	** < 0.001**	**0.043**	**0.106**	**0.0569**	**2.51**	**0.025**		
Path radius	− 0.006	0.010	− 0.63	0.530	− 0.025	0.013					
**Slipped foot**	**0.026**	**0.013**	**2.00**	**0.047**	**0.000**	**0.053**					
Mid-stance onset	0.016	0.016	1.01	0.312	− 0.015	0.047					
Late stance onset	− 0.033	0.017	− 1.92	0.057	− 0.066	0.001					
Foot-mid interaction	− 0.081	0.045	− 1.80	0.073	− 0.170	0.008					
Foot-late interaction	− 0.012	0.024	− 0.48	0.634	− 0.059	0.036					
**ML slip distance (m)**											
**Intercept**	**0.198**	**0.032**	**6.23**	** < 0.001**	**0.135**	**0.260**	**0.3420**	**8.00**	** < 0.001**		
Path radius	− 0.022	0.018	− 1.25	0.214	− 0.058	0.013					
**Slipped foot**	**− 0.094**	**0.024**	**− 3.89**	** < 0.001**	**− 0.142**	**− 0.046**					
Mid-stance onset	− 0.047	0.031	− 1.53	0.128	− 0.108	0.014					
**Late stance onset**	**− 0.119**	**0.031**	**− 3.81**	** < 0.001**	**− 0.180**	**− 0.057**					
Foot-mid interaction	0.049	0.084	0.58	0.563	− 0.118	0.216					
Foot-late interaction	0.056	0.044	1.26	0.209	− 0.032	0.143					
**AP peak slip velocity (m/s)**											
**Intercept**	**0.491**	**0.132**	**3.71**	** < 0.001**	**0.229**	**0.752**	**0.2916**	**11.02**	** < 0.001**		
Path radius	0.134	0.083	1.62	0.108	− 0.030	0.298					
Slipped foot	− 0.082	0.111	− 0.74	0.460	− 0.301	0.137					
**Mid-stance onset**	**0.328**	**0.131**	**2.49**	**0.014**	**0.068**	**0.588**					
**Late stance onset**	**0.596**	**0.143**	**4.16**	** < 0.001**	**0.312**	**0.879**					
Foot-mid interaction	0.678	0.377	1.796	0.075	− 0.069	1.424					
Foot-late interaction	0.257	0.202	1.27	0.206	− 0.143	0.657					
**ML peak slip velocity (m/s)**											
**Intercept**	**0.971**	**0.165**	**5.90**	** < 0.001**	**0.646**	**1.297**	**0.1733**	**2.99**	**0.009**		
Path radius	− 0.174	0.097	− 1.79	0.076	− 0.366	0.018					
**Slipped foot**	**− 0.292**	**0.132**	**− 2.22**	**0.028**	**− 0.553**	**− 0.032**					
Mid-stance onset	0.260	0.164	1.59	0.115	− 0.064	0.585					
Late stance onset	− 0.038	0.169	− 0.22	0.823	− 0.373	0.297					
Foot-mid interaction	− 0.356	0.457	− 0.78	0.437	− 1.258	0.547					
Foot-late interaction	0.067	0.24	0.28	0.779	− 0.407	0.541					

Statistically significant predictors of each outcome are in bold.

### Slip distance

**Figure 5:**
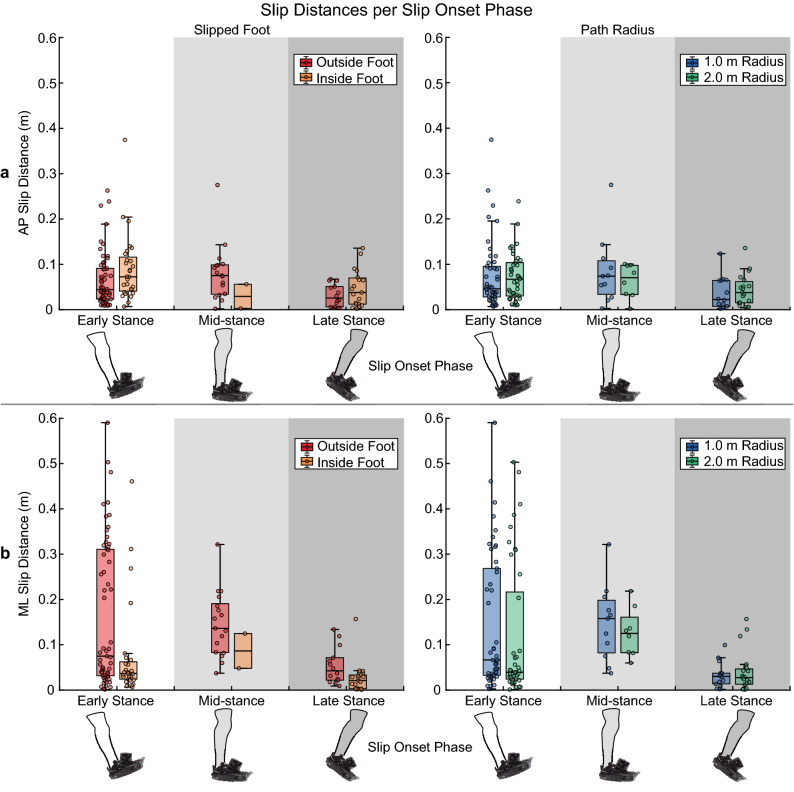
Slip distances in the (**a**) anteroposterior direction and (**b**) mediolateral direction, grouped by slipped foot and path radius. Error bars extend to the most extreme data point within 1.5*IQR of the first and third quartiles, while outliers fall outside the range of 1.5*IQR. No outliers were removed prior to conducting the linear mixed-effects model analysis.

Slip distance in the AP direction was significantly influenced only by the slipped foot (t(132) = 2.00, p = 0.047; Table 2). Inside foot slips tended to travel farther in this direction than outside foot slips (median: 0.06 vs 0.05 m, respectively; Fig. 5a). The mixed effects model significantly predicted AP slip distances, but only accounted for 5.69% of the variance (adjusted R^2^ = 0.0569, F(6, 132) = 2.51, p = 0.025; Table 2). In the ML direction, slip distances were significantly dependent on the slipped foot (t(132) = − 3.89, p < 0.001; Table 2), indicating that those delivered to the inside foot were generally shorter in this direction than those to the outside foot (median: 0.03 vs. 0.08 m, respectively; Fig. 5b). Slips administered in late stance (median: 0.03 m) were also significantly shorter in the ML component compared to those in early stance (median: 0.05 m; t(132) = − 3.81, p < 0.001; Table 2, Fig. 5b). Our model accounted for 34.2% of the variance observed in the ML component of slip distances, and was a significant predictor of this slip attribute (adjusted R^2^ = 0.3420, F(6, 132) = 8.00, p < 0.001; Table 2). Resultant slip distances across the three slip contexts are presented in Supplementary Fig. S5 online.

### Peak slip velocity

**Figure 6:**
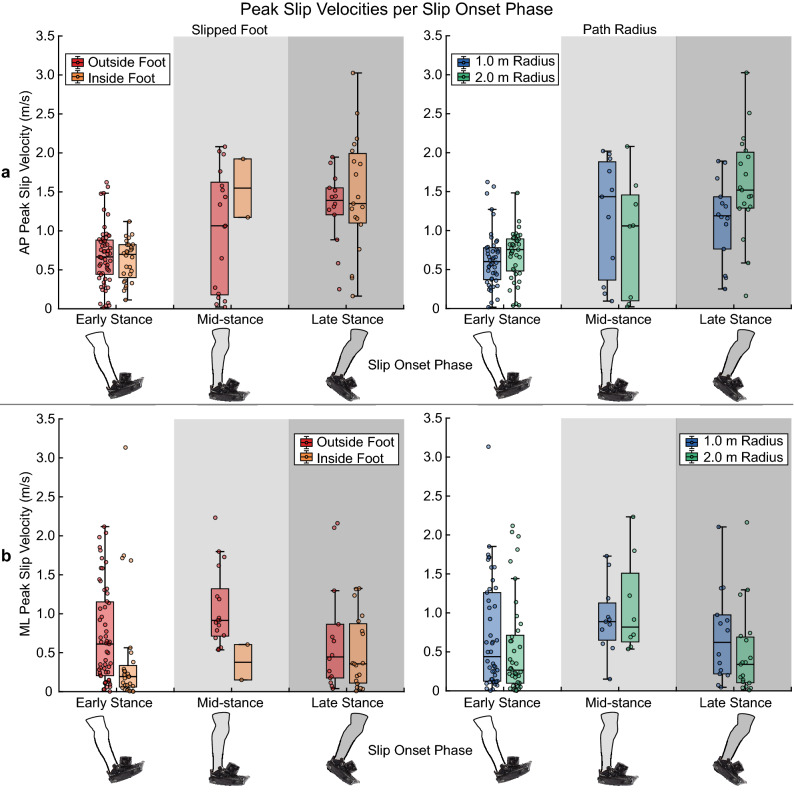
Peak slip velocities in the (**a**) anteroposterior direction and (**b**) mediolateral direction, grouped by slipped foot and path radius. Error bars extend to the most extreme data point within 1.5 * IQR of the first and third quartiles, while outliers fall outside the range of 1.5 * IQR. No outliers were removed prior to conducting the linear mixed-effects model analysis.

In the AP direction, peak slip velocities varied significantly with slip onset phase (mid-stance: t(132) = 2.49, p = 0.014; late stance: t(132) = 4.16, p < 0.001; Table 2), trending faster as slips began later in stance regardless of slipped foot or path radius (median early stance: 0.69 m/s, mid-stance: 1.17 m/s, late stance: 1.35 m/s; Fig. 6a). 29.16% of the variance in AP peak slip velocity was explained by the linear mixed effects model, which significantly predicted this outcome measure (adjusted R^2^ = 0.2916, F(6, 132) = 11.02, p < 0.001; Table 2). Conversely, peak slip velocities in the ML direction were dependent on the slipped foot (t(132) = − 2.22, p = 0.028; Table 2) and not slip onset phase. On average, inside foot slips attained a slower ML peak velocity than outside foot slips (median: 0.22 m/s vs. 0.64 m/s, respectively; Fig. 6b). Our model for ML peak slip velocities was also a significant predictor of this attribute, accounting for 17.33% of the variance in our data (adjusted R^2^ = 0.1733, F(6, 132) = 2.99, p = 0.009; Table 2). Resultant peak slip velocities across the three slip contexts are shown in Supplementary Fig. S5 online.

## Discussion

In the present study, we investigated the effects of various biomechanical contexts on slip mechanics during curvilinear walking. Our protocol was designed to deliver a uniform distribution of slips across stance phase, however successfully delivered slip trials formed a distinctly bimodal distribution that concentrated within early and late stance phases. While our results in total partially support our hypotheses that slip mechanics on a curved trajectory are influenced by context, the degree of influence that each individual context has varies. Slip directions show the greatest reliance on context, with significant effects of slip onset phase, slipped foot, and path radius. The direction that the slipped foot travels relative to the body transitions from primarily anterior to posterior as slip onset occurs later in stance, and whether this transition moves away from or toward/across the body’s midline depends on which foot relative to the path is slipped. In addition, larger path radii are associated with larger angles between the sliding foot’s heading and that of the body (i.e. greater ML components). Slips of the inside foot relative to the path travel slower and a shorter distance in the ML direction but farther in the AP direction compared to those of the outside foot. Finally, later slip onset reduces the ML component of slip distances but increases peak slip velocity in the posterior direction.

Despite our attempt to administer a uniform distribution of slips across stance phase, a disproportionate number of those that actually occurred began during either early or late stance (Table 1), matching the pattern seen during straight walking^22^. Interestingly, successfully delivered slips during mid-stance were nearly exclusive to the outside foot, and over 80% of the failed trials across all phases of stance were targeting the inside foot (Fig. 3). One explanation for this may be that the inside and outside legs relative to the path are fulfilling different functions during curvilinear walking and have different odds of slipping as a result. During curvilinear walking at a comfortable velocity, the CoM is positioned directly above the inside leg^6^, which generates a stronger vertical GRF at mid-stance than the outside leg or during straight walking^30^. In addition, the heading of the CoM during inside leg stance does not change drastically^6^, meaning the centripetal acceleration present at that moment would be minimal. Based on these facts and our findings, the primary role of the inside leg may be to provide body weight support. Conversely, the outside leg may be responsible for changing one’s heading, supported by the “stop sign” CoM trajectory where the apexes align with outside foot steps^6^ and by laterally-directed GRFs and impulses that are of greater magnitude than those of the inside leg or during straight walking^6,7^. These distinct functions likely come with very different friction requirements to prevent a loss of traction. Our results suggest that the required coefficient of friction during inside foot stance is less than that for outside foot stance, resulting in the lack of successfully delivered slips to the inside foot. To our knowledge, however, these measures have not been directly quantified nor compared for curvilinear walking. On this point, it is important to note the distinction between more continuous, curvilinear walking tasks as administered here and transient step or spin turns studied elsewhere^31^. Friction demands have been reported for the latter^8,10^, but we cannot apply their findings to circular paths where the change in direction happens at a constant rate over multiple strides. Therefore, future research should investigate friction demands during curvilinear walking and further examine the specific roles of the inside and outside lower limbs during this task.

Slip directions were influenced by slip onset phase and the slipped foot relative to the path (Fig. 4). Previously reported shear GRFs during curvilinear walking and turning offer a likely explanation for this connection and the trends seen in the data. During curvilinear walking, ML GRFs are elevated, and their direction depends on which foot relative to the path is in stance: ipsilateral for the outside foot, and contralateral for the inside foot^6^. This aligns with the pattern of slip directions we observed in each leg. Similarly, the progression of slip directions from anterior to posterior with advancing onset phase is consistent with braking and propulsive GRFs during turning^7^ and mirrors the relationship seen during straight walking^22^. Slip directions were also impacted by path radius, with slips on the 2.0-m radius path possessing greater ML direction components. A potential reason for this effect is not immediately available from past research, as no published work to our knowledge has compared gait kinetics between curved paths of different radii.

Slip distances and peak velocities were also dependent on both the slipped foot relative to the path and slip onset phase, but not path radius (Figs. 5, 6). Inside foot slips were shorter and slower in the ML direction than outside foot slips, possibly due, again, to the difference in ML GRF magnitudes generated by the inside and outside legs^6^. In the AP direction, however, inside foot slips were slightly longer than outside foot slips on average. In isolation, these findings may lead to the conclusion that inside foot slips during curvilinear walking are less severe than those to the outside foot. This conclusion is not apparent when paired with the direction that these slips are moving, though. Inside foot slips are contralaterally-directed and require a challenging cross-over step with the outside foot in order to recover^15,32–34^, while outside foot slips move ipsilaterally and are countered with an easier lateral step by the inside foot. Because the compensatory stepping demands differ between sides, slip direction should also be accounted for when estimating fall risk. The lack of an effect of path radius on slip distances and peak velocities may stem from our choice to allow participants to walk at a self-selected comfortable speed for each path, which resulted in a slower average walking speed on the 1.0-m radius path than the 2.0-m radius path. Humans progressively slow their walking speed with decreasing path radius when compared to straight walking^11,14,30^, a behavior recently shown to minimize metabolic cost^35^. Slowing gait speed could also diminish falling risk by reducing centripetal force^6,14^, since centripetal force demands would increase as walking trajectories become “tighter” (i.e. smaller radii) if walking velocity is held constant. This centripetal force is generated from ML GRFs; therefore, an increase in the required centripetal force would lead to a concomitant increase in the required coefficient of friction needed to generate such forces without slipping. Indeed, turning at faster gait speeds leads to greater friction requirements^8^. If gait speed reduction is truly a protective mechanism against slipping, then fixing walking speed across both semicircular paths would have resulted in longer and/or faster slips on the 1.0-m radius path than on the 2.0-m radius path. This hypothesis need not be mutually exclusive from metabolic cost minimization, as both functions can be optimized simultaneously^36^. Finally, the linear mixed effects models accounting for slipped foot, slip onset, and path radius only accounted for 5.7–34.2% of the variability in each component of slip distance and peak velocity (Table 2). This suggests that slip distance and velocity may depend more on what happens after a slip begins, such as the individual’s reaction, than on the initial conditions.

Slips during curvilinear walking have been largely omitted from past perturbation research and slip-specific balance training protocols, despite the prevalence of curvilinear gait in daily life^5^ and its accompanying friction demands^8^. Curvilinear walking introduces kinetic asymmetries between inside and outside legs^6,7,11–13^, which our results show has a significant influence on aspects of the slip perturbation that is experienced. Straight, level walking, which has been the focus of past work, does not suffer from the same asymmetries. Therefore, participants in current slip-specific balance training protocols are not exposed to and, as a result, may not develop explicit recovery strategies to the unique conditions that curvilinear walking evokes. The contralaterally-directed slips that we observed following loss of traction under the inside foot, a rare occurrence after unconstrained slips during straight walking^22^, are prime examples. Incorporating curvilinear walking slips into balance training protocols, particularly that target both feet relative to the path and are distributed across stance phase, may reinforce recovery skills that generalize to slips on both straight and curvilinear paths, similar to how improvements stemming from slip-specific training have been shown to generalize to trips^37–39^. Future research should evaluate whether slip-specific training regimens using slips exclusively during straight walking impart any benefit to recovery in curvilinear walking situations, as well as the efficacy of regimens delivering slips during both tasks.

This study is not without limitations. First, we allowed participants to self-select a comfortable walking speed for each path radius, then held them as close as possible to that speed during the slipping trials. This was done to obtain natural gait, slip, and recovery patterns that mimic how they would occur in the environment, rather than placing an unnatural constraint on each participant by forcing them to walk at a slower or faster than normal pace. However, this likely influenced our results as discussed previously. Had we fixed walking speed across all participants and path radii, we may have seen greater differences attributable to path radius. Second, after the first slip exposure, participants likely expected every trial to end with a slip. This may have led to anticipatory gait adjustments that would not be present before an actual, environmental slip. Nevertheless, the exact timing, location, and slipped foot of each instance was still unknown to participants, thereby minimizing the predictability of the perturbation they would face. Finally, we could not objectively discriminate trials that resulted in falls from those that were successful recoveries. This is commonly done using a load cell placed in line with the harness^40^. For our harness system to allow curvilinear walking, we placed enough slack in the ceiling-mounted strap to permit participants to follow the path, yet not enough that their knees would contact the ground in the event of a fall. Because of this slack, attaching a load cell to the strap would have been hazardous to the participant in the harness, and any data obtained from it would be unreliable.

In summary, we demonstrated that slip perturbations delivered on a semicircular path are modified by the contexts present when they begin. The slipped foot relative to the path exerted the greatest influence on slip mechanics, followed by slip onset phase and finally path radius. Much of our results are likely explained by the shear GRFs present throughout stance when following a curvilinear path. In addition, the unbalanced distribution of successfully delivered inside and outside foot slips suggests that the two lower limbs may be fulfilling different functions during curvilinear walking. Next steps should examine the recovery strategies employed by individuals after slipping on a curved path and whether adding such conditions to slip-specific fall prevention methods broadens the recovery skills they impart.

## Supplementary Information

Supplementary Information.

## Data Availability

The datasets generated and analyzed during the current study are available from the corresponding author on reasonable request.
