# Supplementary material for: Slipping mechanics during walking along curved paths depend on the biomechanical context at slip onset
Source: Sci Rep. 2022 Oct 23;12:17801. doi: 10.1038/s41598-022-21701-7 (PMC9588765; doi:10.1038/s41598-022-21701-7)
Supplement: Supplementary file 1 — Supplementary Information. [file 41598_2022_21701_MOESM1_ESM.pdf]

# Slipping Mechanics during Walking Along Curved Paths Depend on the Biomechanical Context at Slip Onset

Corbin M. Rasmussen[1*], Carolin Curtze[1], Mukul Mukherjee[1], and Nathaniel H. Hunt[1]

[1]Department of Biomechanics, University of Nebraska at Omaha, Omaha, NE, USA

# Supplementary Methods

## 1. Tuning the Circular Mixed Effect Model

The circular mixed effects model was created using the R package "bpnreg", which uses an embedding method to generate bivariate estimates of each coefficient in the model equivalent to x- and y-coordinates on the unit circle [1–5]. Because model coefficients are estimated using a Markov Chain Monte Carlo sampler, the number of model iterations, burn-in period (i.e. the first *n* iterations to exclude from the final estimate calculation to only include samples that have converged), and lag (i.e. every $n^{th}$ sample is included in each estimate calculation to prevent autocorrelation between samples) must be determined to ensure all coefficients converge on reliable estimates. To tune the model parameters, five iterations of the circular mixed effects model were run in parallel to generate five different sets of posterior estimates. The outcomes of these five models were then evaluated for convergence by calculating the potential scale reduction factor for each coefficient estimate. Factors near or equal to 1 indicate that additional iterations of the model will not improve convergence and, therefore, that the parameters are sufficient to generate reliable coefficient estimates [6–8]. Multivariate potential scale reduction factors (MPSRF) for the first and second components of each coefficient estimate are presented here to summarize the convergence

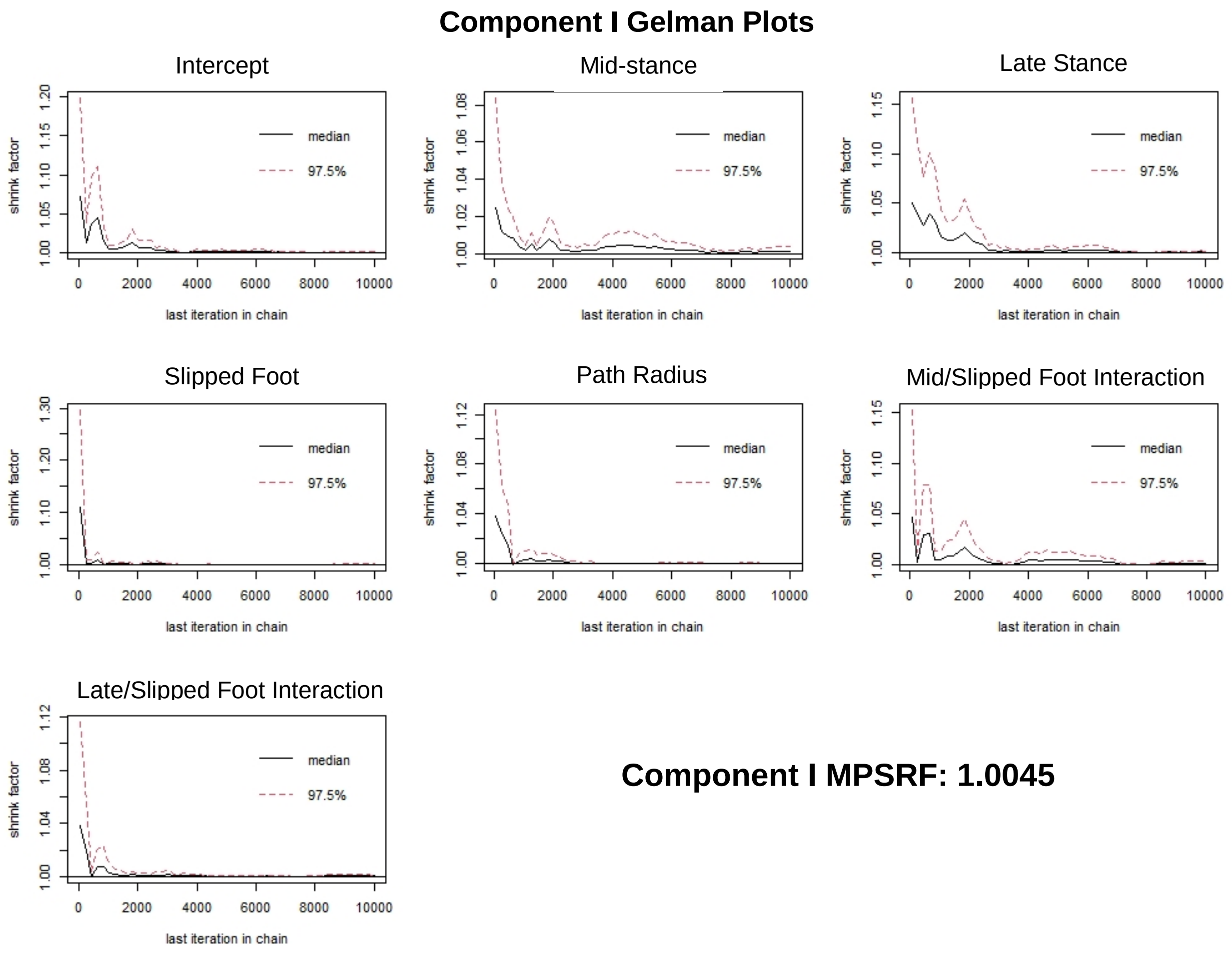


**Supplementary Figure S1:** Gelman plots of the potential scale reduction factor for the first component of each coefficient estimate in the model up to 10,000 model iterations and the MPSRF for the first component at 10,000 iterations, a burn-in period of 2,000 iterations, and lag of 3.

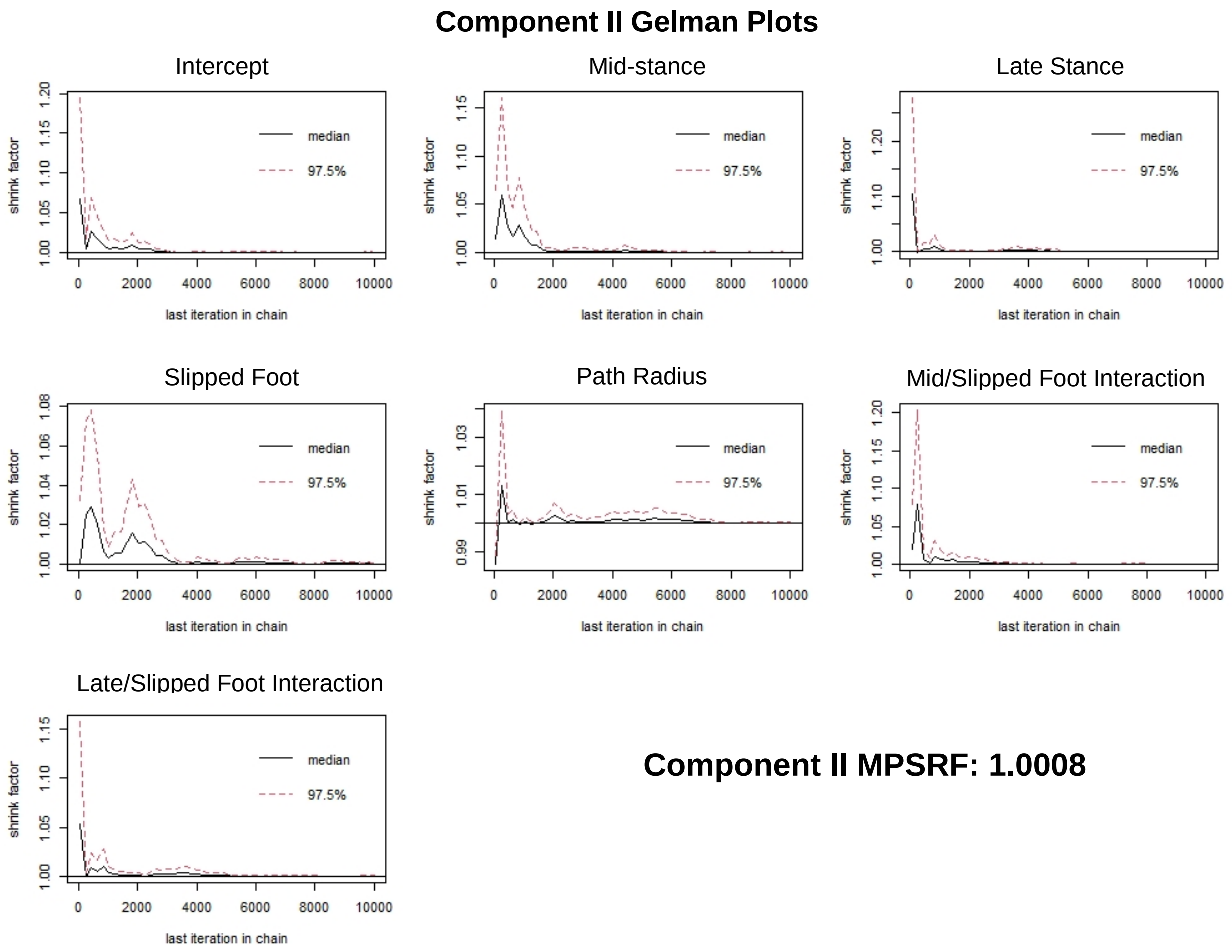


**Supplementary Figure S2:** Gelman plots of the potential scale reduction factor for the second component of each coefficient estimate in the model up to 10,000 model iterations and the MPSRF for the second component at 10,000 iterations, a burn-in period of 2,000 iterations, and lag of 3.

results of the parameters entered into the final model in our analysis. To further assess convergence, Gelman plots were generated for both components of each coefficient estimate (Supplementary Figs. S1 and S2) [8].

After iteratively adjusting the parameters entered into the five parallel models, we settled on 10,000 iterations, a burn-in period of 2,000 iterations, and a lag of 3 for our final circular model used in the analysis. We also entered a seed value of 101 to ensure the final model returned the same results after every execution. The choice of 101 is arbitrary, it only fixes the "starting point" of the model. No seed was entered when tuning the model. Trace plots were generated for both components of each coefficient estimate of the final model to double-check convergence and autocorrelation across iterations (Supplementary Figs. S3 and S4). The desired appearance of a trace plot is a "fat caterpillar"; the values should fluctuate about the coefficient estimate and not stay at an adjacent value over many iterations [1].

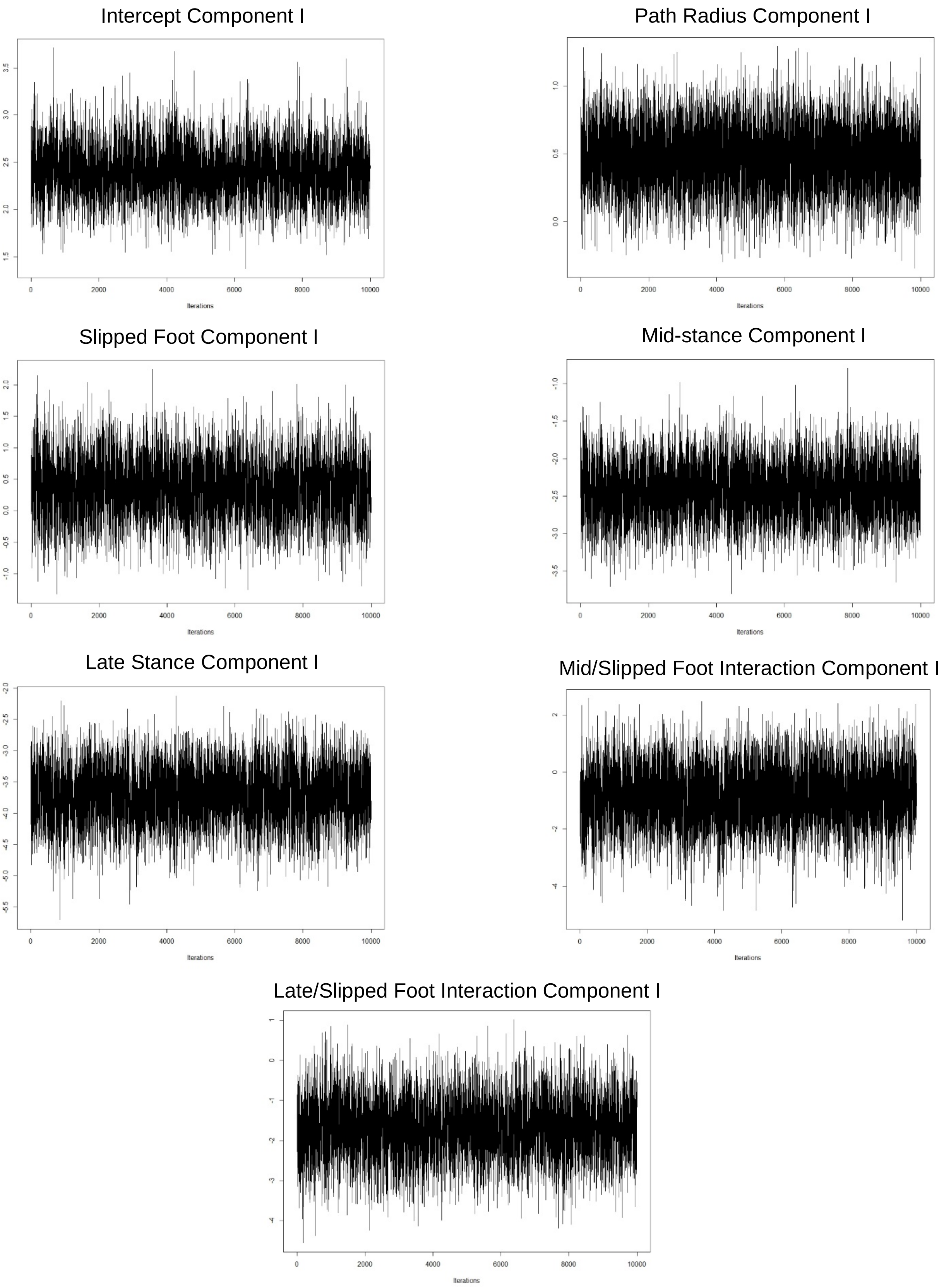


**Supplementary Figure S3:** Trace plots for component I of each coefficient estimate across model iterations.

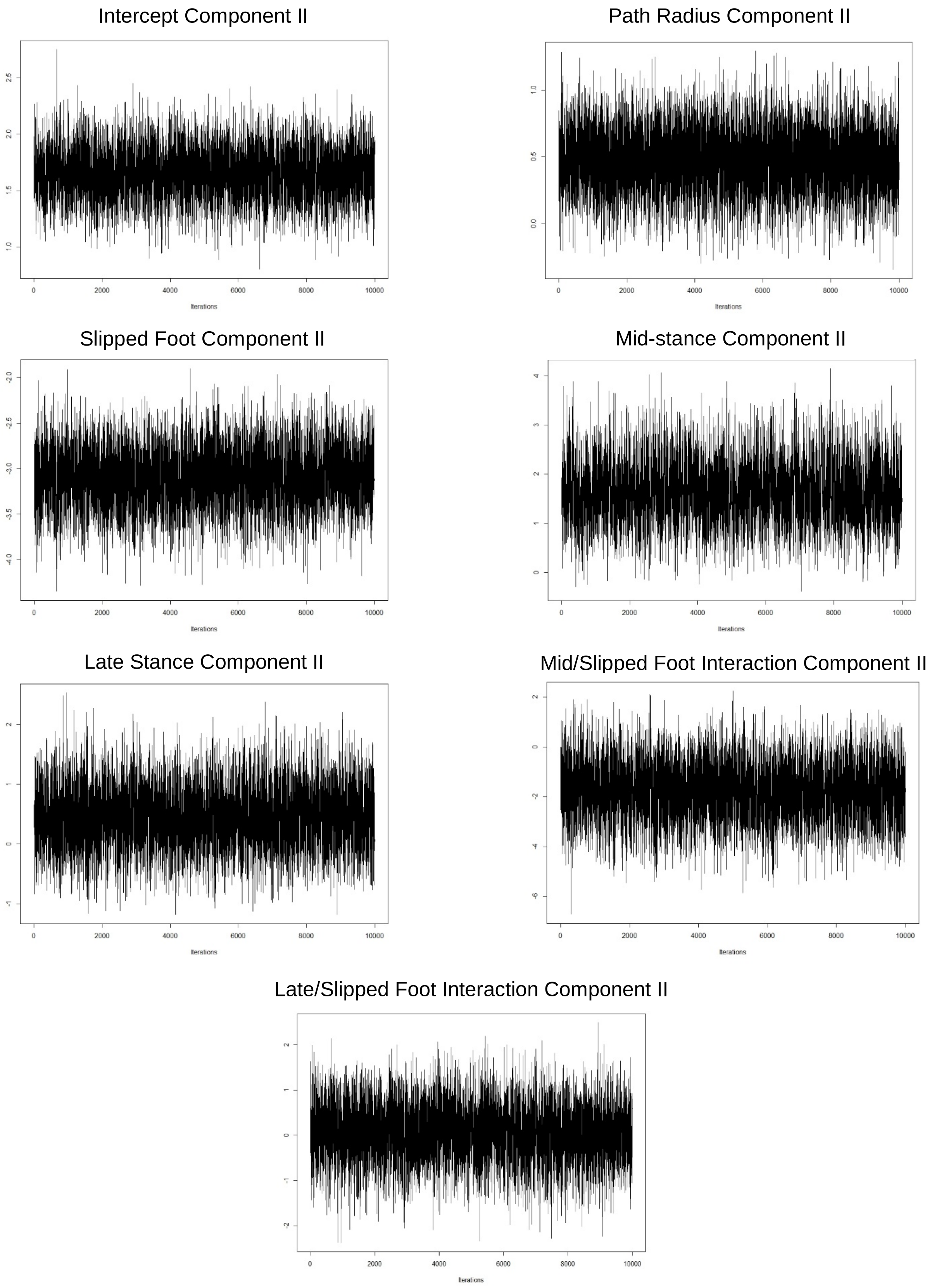


**Supplementary Figure S4:** Trace plots for component II of each coefficient estimate across model iterations.

## 2. Transforming Bivariate Coefficient Estimates

The bivariate coefficient estimates output by the circular mixed effects model are difficult to interpret in terms of direction. Univariate estimates of direction can be derived from the bivariate output of the model since component I and II of each coefficient estimate is analogous to an x- and y-coordinate, respectively. The function "coef_circ" contained within the "bpnreg" R package performs this conversion on all outcome measures of the model[2] using the two-argument arctangent (atan2) function. The atan2 function works as follows [3]:

$$
\theta = \text{atan2}\,(\hat{y}^{II}, \hat{y}^{I}) = \begin{cases}
\tan^{-1}\left(\frac{\hat{y}^{II}}{\hat{y}^{I}}\right) & \text{if } \hat{y}^{I} > 0; \\
\tan^{-1}\left(\frac{\hat{y}^{II}}{\hat{y}^{I}}\right) + \pi & \text{if } \hat{y}^{I} < 0,\ \hat{y}^{II} \geq 0; \\
\tan^{-1}\left(\frac{\hat{y}^{II}}{\hat{y}^{I}}\right) - \pi & \text{if } \hat{y}^{I} < 0,\ \hat{y}^{II} < 0; \\
\frac{\pi}{2} & \text{if } \hat{y}^{I} = 0,\ \hat{y}^{II} > 0; \\
-\frac{\pi}{2} & \text{if } \hat{y}^{I} = 0,\ \hat{y}^{II} < 0; \\
\text{undefined} & \text{if } \hat{y}^{I} = 0,\ \hat{y}^{II} = 0.
\end{cases}
$$

The atan2 function returns univariate estimates of direction in a range of -180°-180°. Because we present our results in a range of 0°-360°, negative direction values were further converted to their positive counterparts by adding 360° to the negative value.

## 3. References


1. Cremers, J. & Klugkist, I. One direction? a tutorial for circular data analysis using R with examples in cognitive psychology. *Front. Psychol.* **9**, 2040 (2018).

2. Cremers, J. bpnreg: bayesian projected normal regression models for circular data. (2020).

3. Cremers, J., Mulder, K. T. & Klugkist, I. Circular interpretation of regression coefficients. *Br. J. Math. Stat. Psychol.* **71**, 75–95 (2018).

4. R Core Team. R: a language and environment for statistical computing. (2021).

5. Cremers, J., Mainhard, T. & Klugkist, I. Assessing a bayesian embedding approach to circular regression models. *Methodology* **14**, 69–81 (2018).

6. Brooks, S. P. & Gelman, A. General methods for monitoring convergence of iterative simulations. *J. Comput. Graph. Stat.* **7**, 434–455 (1998).

7. Gelman, A. *et al. Bayesian Data Analysis*. (Chapman and Hall/CRC, 2013).

8. Gelman, A. & Rubin, D. B. Inference from iterative simulation using multiple sequences. *Stat. Sci.* **7**, 457–511 (1992).

# Supplementary Figures

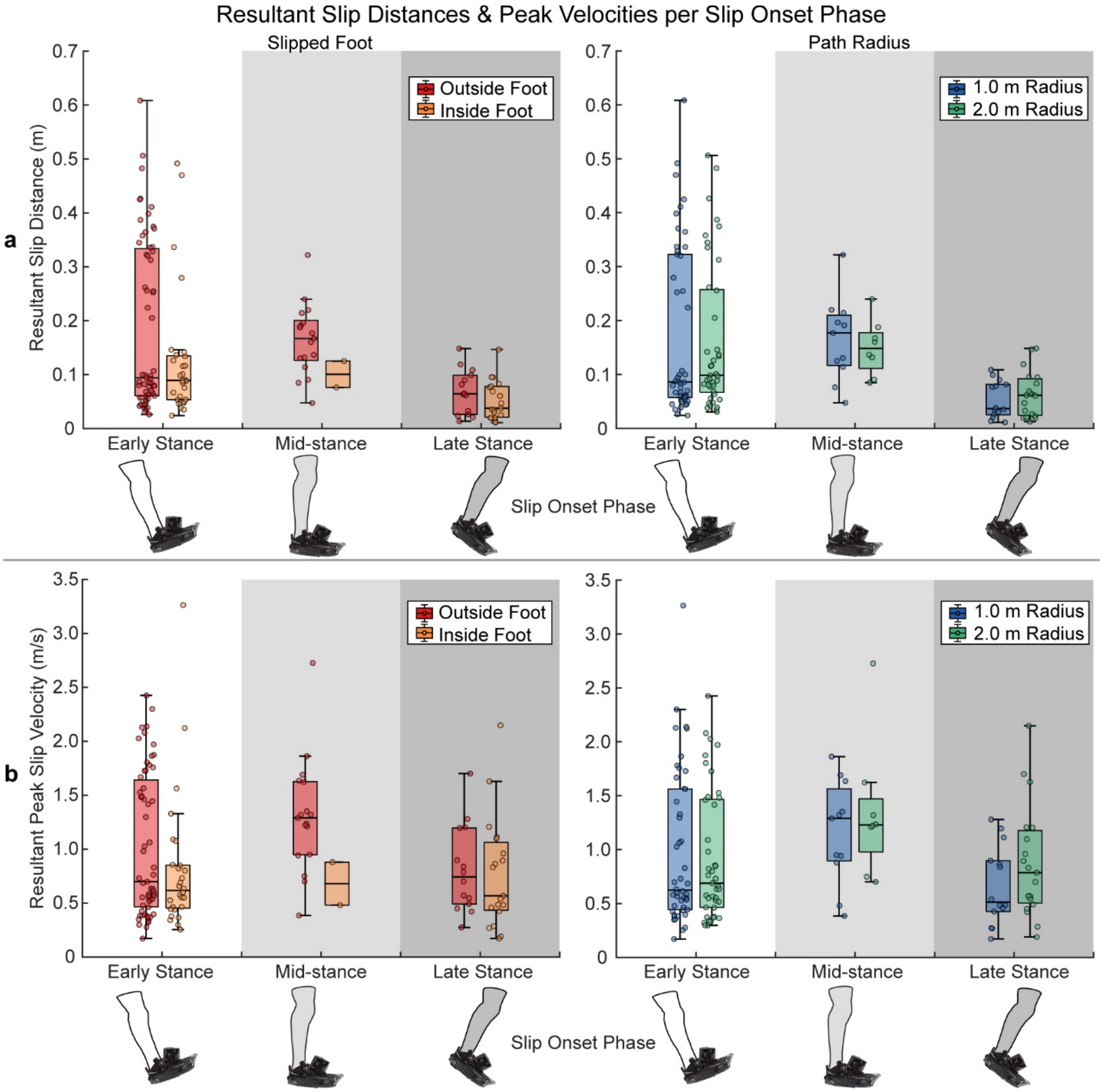


**Supplementary Figure S5:** Resultant **a)** slip distances and **b)** peak slip velocities, grouped by slipped foot and path radius. Error bars extend to the most extreme data point within 1.5*IQR of the first and third quartiles, while outliers fall outside the range of 1.5*IQR.

STROBE Statement—Checklist of items that should be included in reports of ***cross-sectional studies***

| Topic | Item | Recommendation | Location |
|---|---|---|---|
| **Title and Abstract** | 1 | *a)* Indicate the study's design with a commonly used term in the title or the abstract | Abstract Sent. 3 |
| | | *b)* Provide in the abstract an informative and balanced summary of what was done and what was found | Abstract Sent. 4-9 |
| **Introduction** | | | |
| Background/Rationale | 2 | Explain the scientific background and rationale for the investigation being reported | Intro. Para. 1-3 |
| Objectives | 3 | State specific objectives, including any prespecified hypotheses | Intro. Para. 4 |
| **Methods** | | | |
| Study Design | 4 | Present key elements of study design early in the paper | Sec. 2.3, 2.4 |
| Setting | 5 | Describe the setting, locations, and relevant dates, including periods of recruitment, exposure, follow-up, and data collection | - |
| Participants | 6 | Give the eligibility criteria, and the sources and methods of selection of participants | Sec. 2.1 |
| Variables | 7 | Clearly define all outcomes, exposures, predictors, potential confounders, and effect modifiers. Give diagnostic criteria, if applicable | Sec. 2.4, 2.5 |
| Data Sources/ Measurement | 8* | For each variable of interest, give sources of data and details of methods of assessment (measurement). Describe comparability of assessment methods if there is more than one group | Sec. 2.3-2.5 |
| Bias | 9 | Describe any efforts to address potential sources of bias | Sec. 2.4 |
| Study Size | 10 | Explain how the study size was arrived at | - |
| Quantitative Variables | 11 | Explain how quantitative variables were handled in the analyses. If applicable, describe which groupings were chosen and why | Sec. 2.5 |
| Statistical Methods | 12 | *a)* Describe all statistical methods, including those used to control for confounding | Sec. 2.6 |
| | | *b)* Describe any methods used to examine subgroups and interactions | Sec. 2.6 |
| | | *c)* Explain how missing data were addressed | Sec. 2.6 |
| | | *d)* If applicable, describe analytical methods taking account of sampling strategy | - |
| | | *e)* Describe any sensitivity analyses | Sec. 2.6 |
| **Results** | | | |
| Participants | 13* | *a)* Report numbers of individuals at each stage of study—eg numbers potentially eligible, examined for eligibility, confirmed eligible, included in the study, completing follow-up, and analysed | Sec. 2.1 |
| | | *b)* Give reasons for non-participation at each stage | N/A |
| | | *c)* Consider use of a flow diagram | - |
| Descriptive Data | 14* | *a)* Give characteristics of study participants (eg demographic, clinical, social) and information on exposures and potential confounders | Sec. 2.1 |
| | | *b)* Indicate number of participants with missing data for each variable of interest | N/A |
| Outcome Data | 15* | Report numbers of outcome events or summary measures | Sec. 3.1, Tab. 1, Fig. 3 |
| Main Results | 16 | *a)* Give unadjusted estimates and, if applicable, confounder-adjusted estimates and their precision (eg, 95% confidence interval). Make clear which confounders were adjusted for and why they were included | Sec. 3.2-3.4, Table 2 |
| | | *b)* Report category boundaries when continuous variables were categorized | N/A |
| | | *c)* If relevant, consider translating estimates of relative risk into absolute risk for a meaningful time period | N/A |
| Other Analyses | 17 | Report other analyses done—eg analyses of subgroups and interactions, and sensitivity analyses | Sec. 3.3, 3.4 |
| **Discussion** | | | |
| Key Results | 18 | Summarise key results with reference to study objectives | Discussion Para. 1 |
| Limitations | 19 | Discuss limitations of the study, taking into account sources of potential bias or imprecision. Discuss both direction and magnitude of any potential bias | Discussion Para. 6 |
| Interpretation | 20 | Give a cautious overall interpretation of results considering objectives, limitations, multiplicity of analyses, results from similar studies, and other relevant evidence | Discussion Para. 2-5 |
| Generalisability | 21 | Discuss the generalisability (external validity) of the study results | Discussion Para. 5 |
| **Other Information** | | | |
| Funding | 22 | Give the source of funding and the role of the funders for the present study and, if applicable, for the original study on which the present article is based | Acknowledgements |

*Give information separately for exposed and unexposed groups
